# Autologous platelet-rich plasma induces bone formation of tissue-engineered bone with bone marrow mesenchymal stem cells on beta-tricalcium phosphate ceramics

**DOI:** 10.1186/s13018-017-0665-1

**Published:** 2017-11-21

**Authors:** Tengbo Yu, Huazheng Pan, Yanling Hu, Hao Tao, Kai Wang, Chengdong Zhang

**Affiliations:** 1 Department of Orthopaedic Surgery, Affiliated Hospital of Qingdao University, Qingdao, Shandong 266000 People’s Republic of China; 2 Department of Clinical Laboratory, Affiliated Hospital of Qingdao University, Qingdao, Shandong 266000 People’s Republic of China

**Keywords:** Platelet-rich plasma, Tissue-engineered bone, Beta-tricalcium phosphate, Osteogenic, Scaffold, Autologous

## Abstract

**Background:**

The purpose of the study is to investigate whether autologous platelet-rich plasma (PRP) can serve as bone-inducing factors to provide osteoinduction and improve bone regeneration for tissue-engineered bones fabricated with bone marrow mesenchymal stem cells (MSCs) and beta-tricalcium phosphate (β-TCP) ceramics. The current study will give more insight into the contradictory osteogenic capacity of PRP.

**Methods:**

The concentration of platelets, platelet-derived growth factor-AB (PDGF-AB), and transforming growth factor-β1 (TGF-β1) were measured in PRP and whole blood. Tissue-engineered bones using MSCs on β-TCP scaffolds in combination with autologous PRP were fabricated (PRP group). Controls were established without the use of autologous PRP (non-PRP group). In vitro, the proliferation and osteogenic differentiation of MSCs on fabricated constructs from six rabbits were evaluated with MTT assay, alkaline phosphatase (ALP) activity, and osteocalcin (OC) content measurement after 1, 7, and 14 days of culture. For in vivo study, the segmental defects of radial diaphyses of 12 rabbits from each group were repaired by fabricated constructs. Bone-forming capacity of the implanted constructs was determined by radiographic and histological analysis at 4 and 8 weeks postoperatively.

**Results:**

PRP produced significantly higher concentration of platelets, PDGF-AB, and TGF-β1 than whole blood. In vitro study, MTT assay demonstrated that the MSCs in the presence of autologous PRP exhibited excellent proliferation at each time point. The results of osteogenic capacity detection showed significantly higher levels of synthesis of ALP and OC by the MSCs in combination with autologous PRP after 7 and 14 days of culture. In vivo study, radiographic observation showed that the PRP group produced significantly higher score than the non-PRP group at each time point. For histological evaluation, significantly higher volume of regenerated bone was found in the PRP group when compared with the non-PRP group at each time point.

**Conclusions:**

Our study findings support the osteogenic capacity of autologous PRP. The results indicate that the use of autologous PRP is a simple and effective way to provide osteoinduction and improve bone regeneration for tissue-engineered bone reconstruction.

## Background

Repair of bone defects remains a difficult challenge in orthopedic and maxillofacial surgery. A variety of strategies have been developed based on the dogma that the effectiveness of any successful bone graft material generally can be attributed to one or more of three properties: osteogenic cells, osteoconduction, and osteoinduction [1].

Nowadays, a tissue engineering strategy has appeared as one of the most promising approaches in regenerating damaged or diseased bones [2, 3]. The challenges of bone tissue engineering emphasize the need to provide osteoblast-like cells combined with growth factors in a biomechanically stable scaffold. Bone marrow mesenchymal stem cells (MSCs) have been demonstrated to be a very attractive cell source in tissue engineering due to their capacity to differentiate into lineages of the mesenchymal tissues, including the bone, the cartilage, and the muscle [4]. With regard to the scaffold used in bone tissue engineering, beta-tricalcium phosphate (β-TCP) ceramics have been extensively recognized as scaffolds because they possess satisfactory biocompatibility, osteoconductivity, and mechanical properties [5, 6]. However, they do not have osteoinductive capacity. This incapacity of inducing bone formation can be overcome by adding growth factors and cytokines onto ceramic scaffolds.

Platelet-rich plasma (PRP) is a concentration of platelets in a small volume of plasma. Because biological factors released by platelets have an effect on osteo-competent cells, scientists have proposed the delivery of a concentrate of platelets at the site of the injury as a successful strategy for fostering the regeneration pathway during bone wound healing [7, 8]. Autologous PRP has been used for the treatment of bone defects in maxillofacial surgery and orthopedics for accelerating bone formation [9, 10]. However, the effects of PRP on the enhancement of bone regeneration remain debated. Some studies have not observed any improvement in bone formation and maturation [11–14]. The contradictory PRP study outcomes remain to be elucidated. Furthermore, to the best of our knowledge, there are no studies that evaluated the performance of autologous PRP in combination with MSCs and β-TCP to repair segmental bone defects in vivo.

In the present study, a tissue-engineered bone using MSCs on a β-TCP scaffold in combination with autologous PRP was developed. The vitro proliferation, osteogenic differentiation of MSCs and in vivo bone-forming capacity in segmental bone defect models of the fabricated constructs were evaluated when in combination with autologous PRP or not.

The purpose of this study is to investigate whether autologous PRP can serve as bone-inducing factors to provide osteoinduction and improve bone regeneration for tissue-engineered bones fabricated with MSCs and β-TCP ceramics. The current study will give more insight into the contradictory osteogenic capacity of PRP.

## Methods

### Animals and scaffold materials

Ten-month-old male New Zealand white rabbits weighing 2.0–2.5 kg were employed. The present research was approved by the Qingdao University Medical College Medical Ethics Committee. All experimental procedures were in compliance with the principles of International Laboratory Animal Care and with the European Communities Council Directive (86/809/EEC).

β-TCP scaffolds (Bio-lu Bio Materials Company Limited, China) were formed into cuboids (5 × 5 × 5 mm) for in vitro study and cylinders (diameter, 4 mm; length, 12 mm) for in vivo study. The scaffold had a high degree of porosity (75 ± 10%) and was completely interconnected. The average pore diameter was 530 ± 50 μm, and the interconnecting channels were 150 ± 50 μm in diameter. These parameters meet the criteria to act as scaffold material for bone regeneration [15, 16].

### Isolation, expansion, and osteogenic differentiation of rabbit MSCs

Samples of MSCs were obtained from iliac bone marrow aspirates of New Zealand white rabbits. MSCs isolation and expansion were performed as reported by Liu H et al. [17]. Briefly, nucleated cells were isolated using the density gradient centrifugation method. Then, cells were cultured at 37 °C in a humidified atmosphere containing 5%CO_2_, and medium was changed every 3 days. Upon reaching about 90% confluence, the cells were trypsinized and replated at 1 × 10^4^ cells/cm^2^ for further expansion. Cell passaging was performed and cells from the third passage were used in the following experiments. The MSCs from rabbits were cultured individually.

To induce osteogenic differentiation, the third passage cells were then cultured for up to 2 weeks in an osteogenic induction medium containing basic culture medium, with the addition of 100 nM dexamethasone, 50 μg/ml ascorbic acid-2-phosphate, and 10 mM β-glycerophosphate (all from Sigma). The culture medium was changed every 3 days. The osteogenic differentiation of MSCs was confirmed by positive results of alkaline phosphatase (ALP) and Alizarin red S staining.

### Preparation of autologous PRP

Autologous PRP was prepared from the same rabbit as used for MSCs isolation as described by Ishida et al. [18]. Briefly, 10 ml of peripheral blood was drawn, via puncture of the central auricular artery, into a syringe containing 1 ml of acid citrate dextrose. The blood sample was initially centrifuged for 15 min at 800 rpm at 20 °C to separate the plasma containing the platelets from the red cells. The plasma was drawn off the top and centrifuged again for 15 min at 2000 rpm at 20 °C to separate the platelets. The platelet-poor plasma was then drawn off the top, leaving the PRP and buffy coat. Then, the buffy coat and PRP were re-suspended, and about 1 ml of PRP was produced.

### In vitro study

Six New Zealand white rabbits were used for in vitro study. The undifferentiated MSCs and their autologous PRP were prepared from these rabbits.

#### Measurement of platelets and growth factors

The concentrations of platelets in PRP and whole blood were counted with an automated hematology analyzer (Nikon, Japan). To measure the concentrations of growth factors in PRP, thrombin (0.8IUactivity)-calcium chloride (1 M) solution (1:1) (Tissucol-Duo, Baxter, Germany) is used to activate the platelets. The fibrin gel was formed within 8 to 10 mins. The concentrations of platelet-derived growth factor-AB (PDGF-AB) and transforming growth factor-β1 (TGF-β1) secreted from activated PRP and whole blood were measured with commercial enzyme-linked immunosorbent assay (ELISA) kit (Cusabio Biotech Co. Ltd., China), according to the manufacturer’s instructions.

#### Cell seeding and in vitro cultivation of constructs

The autologous PRP and cell suspension at a density of 1 × 10^7^ cells/ml were mixed at a volume ratio of 4:1 to obtain cell-PRP mixture at a density of 2 × 10^6^ cells/ml as reported by Tajima et al. [19]. One hundred microliters of cell-PRP mixture was evenly dripped onto each β-TCP scaffold (PRP group). Controls were established without the use of PRP. MSC suspensions at a density of 2 × 10^6^ cells/ml were seeded into β-TCP scaffolds to fabricate constructs (non-PRP group). The constructs were incubated at 37 °C for 2 h to allow for cell diffusion and attachment. Then, 20 μl of thrombin (0.8IUactivity)-calcium chloride (1 M) solution (1:1) (Tissucol-Duo, Baxter, Germany) was added onto fabricated constructs. All constructs were cultured statically for up to 2 weeks in an osteogenic induction medium which contained basic culture medium, with the addition of 100 nM dexamethasone, 50 μg/ml ascorbic acid-2-phosphate, and 10 mM β-glycerophosphate (all from Sigma). The medium was changed every 3 days.

#### Cell proliferation assay

The proliferation of MSCs on fabricated constructs was measured by using the MTT (3-(4,5-dimethylthiazol-2yl)-2,5-diphenyl-2H-tetrazolium bromide) assay after 1, 7, and 14 days of culture as described by Wang H et al. [20]. The absorbance of the solution in each well was measured by a microplate reader (Bio-Tek, USA) at a wavelength of 570 nm.

#### Cell osteogenic differentiation assay

ALP and osteocalcin (OC) as the quantitative indices of osteogenic capability were measured after 1, 7, and 14 days of culture. To assess the ALP activity, cell lysates were prepared using the procedure described by Jiang et al. [21]. ALP activity assay is based on the conversion of p-nitrophenyl phosphate (P-NPP) into p-nitrophenol (P-NP) in the presence of ALP, where the rate of P-NP production is proportional to ALP activity. Protein concentrations of the cell lysates were determined by Bradford protein assay (Bio-Rad Laboratories, USA). The results of ALP activity were expressed as nmol/min/mg protein. OC content was measured by radioimmunoassay using an OC RIA Kit (Dongya, Beijing, China) in accordance with the manufacturer’s instruction. OC content was normalized to protein concentration.

### In vivo study

Twenty-four New Zealand white rabbits used for in vivo study were randomized into the PRP group and non-PRP group by coin flipping. Both groups were then randomly subdivided into two subgroups that were sacrificed after 4 and 8 weeks postoperatively. The osteogenically differentiated MSCs and their autologous PRP were obtained from these rabbits.

#### Preparation of implants

Following the above-mentioned cell seeding method, 200 μl of cell-PRP mixture or cell suspension was respectively dripped onto each cylindrical scaffold. Two hours later, 40 μl of thrombin (0.8IUactivity)-calcium chloride (1 M) solution (1:1) (Tissucol-Duo, Baxter, Germany) was added onto fabricated constructs.

#### Surgical procedures for implantation

The segmental bone defect model was prepared as described by Niemeyer P et al. [22]. Briefly, under sterile conditions, the left radial shaft was exposed through a 2-cm longitudinal incision over the radius. A 12-mm bone segment with periosteum was excised from the middle of the radial diaphysis. The ulna was left intact for mechanical stability. The fabricated constructs were implanted into bone defects according to the scheduled grouping. The middle point of the length for each implanted construct was labeled with a 3–0 suture for the accurate selection of histological sections of specimens. All rabbits were kept in separate cages, fed a standard diet, and allowed to move freely after surgery without plaster immobilization.

#### Radiographic evaluation

Six rabbits from each subgroup were subject to X-ray examination respectively at 4 and 8 weeks postoperatively. The exposure conditions were 45 kV, 125 mA, and 32 ms. The exposure distance was 100 cm. The films were analyzed and scored following Yang’s method [23]. The scale was composed of four categories (Table 1). The proximal, central, and distal part of graft was scored individually, and a summarized score was calculated. Scoring was assessed under a double blinding protocol by two independent observers.

**Table 1:** Radiographic grading system [23]

Grading items	Score
Periosteal reaction^a^	
None	0
Minimal (localized to the gap)	1
Medium (extends over the gap; < 1/4)	2
Moderate (< 1/2 but > 3/4)	3
Full	4
Osteotomy site^a^	
Osteotomy line completely radiolucent	0
Osteotomy line partially radiolucent	2
Osteotomy line invisible	4
Remodeling^a^	
None apparent	0
Intramedullary space	1
Intracortical	2
Graft appearance^a^	
Unchanged/intact	0
Mild resorption	1
Moderate replacement	2
Mostly replaced	3
Fully reorganized	4

^a^Proximal, distal, and central part of graft scored individually

#### Histological evaluation

After the above-mentioned examination, the six rabbits from each subgroup were sacrificed. The implant site (12 mm) along with the host bone (5 mm from either side) of each animal was resected out from the whole bone. The specimens were fixed with 4% buffered paraformaldehyde, decalcified in 50 mM ethylenediaminetetraacetic acid, and embedded in paraffin. The sections (5 μm thick) at the interface region were longitudinally cut and stained with hematoxylin and eosin (HE) to evaluate bony union between host bone and implants. Furthermore, three levels of sections (5 μm thick) were transversely sliced at a quarter, half, and three-quarter of the length of each implanted construct and stained with HE to analyze new bone formation. Each section was observed under light microscope by two independent observers, and 10 images were randomly obtained in one section. All images were transferred to a computer for image processing and analysis (Image-Pro Plus software, Media Cybernetics, USA). The tissue composition of the new bone, β-TCP, and connective tissue were presented as the percentage of bone area in relation to the view field area of each image. Based on the percentage of tissue composition of three representative sections from three levels, the average percentage per implanted construct was calculated.

### Statistical analysis

Results are expressed as mean ± standard deviation. Statistical analysis to compare results between two groups was carried out by independent sample *t* test with SPSS for Windows 15.0 (SPSS, USA). Differences were considered to be significant if *p* < 0.05.

## Results

### Concentration of platelets and growth factors

The mean platelet count for PRP was 1056 ± 106 × 10^3^ platelets/μl, while that of whole blood was 201 ± 23 × 10^3^ platelets/μl (a 5.25-fold increase in platelets in PRP than in whole blood). Correspondingly, PRP produced 2.64-fold increase in PDGF-AB concentration and 3.54-fold increase in TGF-β1 concentration respectively. These values confirmed the presence of a sufficient concentration of platelets during the PRP preparation. The concentration of platelet, TGF-β1, and PDGF-AB were significantly higher in PRP than those in whole blood (*p* < 0.05, Table 2).

**Table 2:** Concentration of platelets and growth factors in PRP and whole blood (*n* = 6, mean ± SD)

	Platelet(10^9^/L)	PDGF-AB(ng/ml)	TGF-β1 (ng/ml)
Whole blood	201.57 ± 23.35	19.43 ± 0.92	36.32 ± 3.87
PRP	1056.28 ± 106.17*	51.25 ± 5.46*	128.62 ± 13.05*

*p < 0.05 compared with whole blood

### Cell proliferation in vitro

**Fig. 1:**
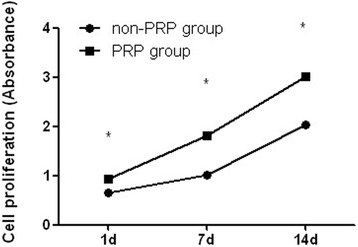
Cell proliferation in vitro. The PRP group demonstrated significantly higher absorbance values compared with the non-PRP group at each time point. (Data in mean ± SD, *n* = 6, *p < 0.05)

The absorbance values for two groups of MSCs increased with the length of the culture period. The PRP group demonstrated significantly higher absorbance values compared with the non-PRP group at all time points (*p* < 0.05), which indicates that autologous PRP has a stimulative effect on the MSCs proliferation (Fig. 1).

### Cell osteogenic differentiation in vitro

**Fig. 2:**
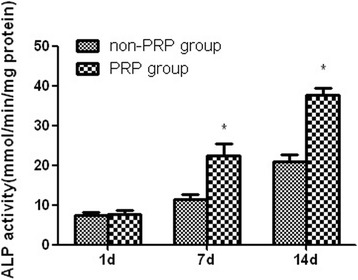
ALP activity for cell osteogenic differentiation in vitro. Significantly higher ALP activity was detected for the PRP group when compared to that for the non-PRP group at 7 and 14 days after seeding. (Data in mean ± SD, *n* = 6, *p < 0.05)

**Fig. 3:**
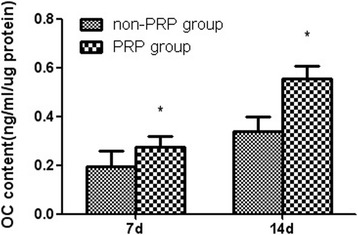
OC content for cell osteogenic differentiation in vitro. The PRP group exhibited significantly higher OC content than the non-PRP group on days 7 and 14 after being cultured. (Data in mean ± SD, *n* = 6, *p < 0.05)

The ALP activity of the two groups of MSCs increased with prolonged incubation period. At 1 day after seeding, there was no significant difference in ALP activity. However, significantly higher ALP activity was detected for the PRP group when compared to the non-PRP group at 7 and 14 days after seeding (*p* < 0.05, Fig. 2). As for OC content, no OC was detected for both groups at 1 day of cultivation. The PRP group exhibited significantly higher OC content than the non-PRP group on days 7 and 14 after being cultured (*p* < 0.05, Fig. 3). These results indicate that autologous PRP is effective for promoting osteogenic differentiation of MSCs.

### Radiographic evaluation

**Fig. 4:**
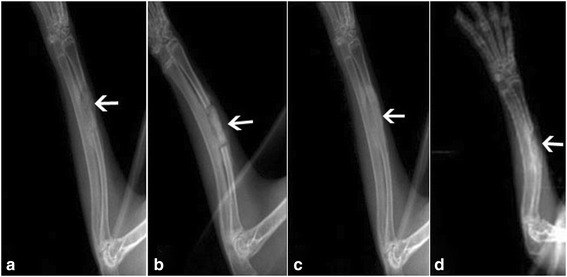
Representative radiographs of critical-sized bone defects repair by PRP group constructs (**a**, **c**) and Non-PRP group constructs (**b**, **d**) at 4 weeks (**a**, **b**) and 8 weeks (**c**, **d**) postoperatively. At 4 weeks postoperatively, the radioopaque areas of implants and radiolucent zone at the interface between the implant and host bone was visible in both groups. However, the boundary of constructs in the PRP group became more cloudy. At 8 weeks postoperatively, a decrease in radiopacity related to the new bone formation and material degradation was more obvious in the PRP group. The radiolucent zone at the interfacial area almost disappeared in the PRP group. The arrow indicates the implanted construct

At 4 weeks postoperatively, the high-density radioopaque areas of implants were clearly identified at bone defect sites in both groups and a distinct radiolucent zone at the interface between the implant and the host bone was visible. However, the boundary of implanted constructs in PRP group became more cloudy along with degradation and more bone callus at the interfacial area was found. At 8 weeks postoperatively, the absence of this radiolucent zone was observed for both groups, which is considered as the union between the implant and host bone. PRP group achieved more osseous callus around the periphery of the implant (Fig. 4). The radiographic grading score at the proximal, central, and distal part of implants graft increased with implantation time in both groups. The PRP group showed significantly higher score than the non-PRP group at each time point (*p* < 0.05, Table 3). Correspondingly, summarized scores showed similar results, also reaching statistical significance (*p* < 0.05, Table 3).

**Table 3:** X-ray scores at the proximal, central and distal location and summarized scores of implants (*n* = 6, mean ± SD)

Group	Time	Proximal	Central	Distal	summarized
Non-PRP group	4 weeks	2.667 ± 0.817	1.5 ± 1.049	3 ± 0.894	7.167 ± 1.835
PRP group	4 weeks	4.833 ± 1.472*	3 ± 0.894*	4.667 ± 1.211*	12.5 ± 2.811*
Non-PRP group	8 weeks	7.5 ± 1.049	3.667 ± 1.633	7 ± 1.549	18.167 ± 1.602
PRP group	8 weeks	10.5 ± 1.653*	6 ± 1.265*	11.833 ± 2.927*	28.333 ± 1.862*

*p < 0.05 compared with the non-PRP group

### Gross view and histological evaluation

All rabbits survived until the scheduled date of sacrifice without any evidence of inflammation or infection at the implantation site such as incision infection or skin necrosis. At 4 weeks after surgery, the entire implanted material could be identified for both groups, and the interface between the implant and the host bone was visible. At 8 weeks postoperatively, both groups achieved integration between implanted constructs and host bone on either side. The implanted material was still identifiable in the middle region of defect in Non-PRP group, while no apparent remnants of implanted material were found in PRP group.

**Fig. 5:**
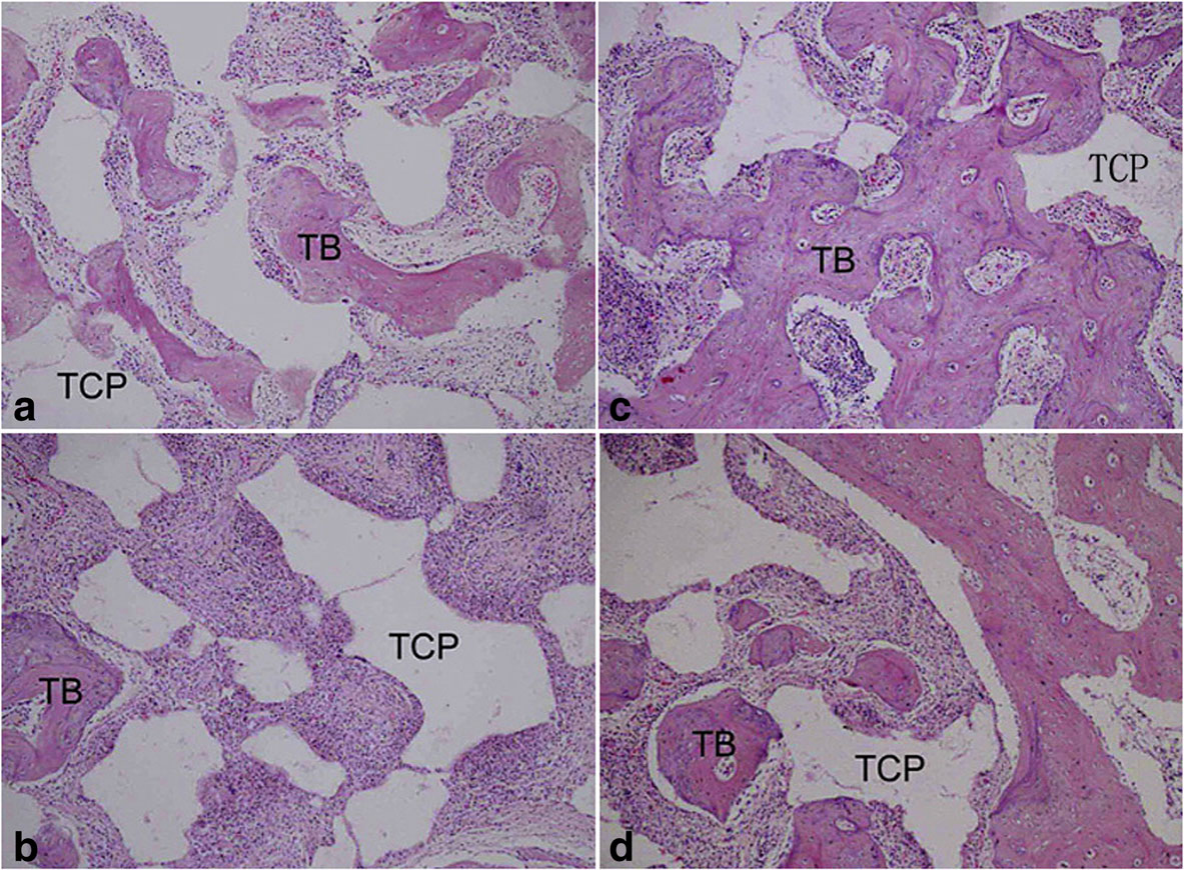
Histological evaluation of regenerated bone of PRP group constructs (**a**, **c**) and non-PRP group constructs (**b**, **d**) at 4 weeks (**a**, **b**) and 8 weeks (**c**, **d**) postoperatively. The PRP group demonstrated more extensive bone formation with the degradation of implanted constructs than the non-PRP group at each time point. (HE staining × 100. TCP, tricalcium phosphate; TB, tissue-engineered bone)

**Fig. 6:**
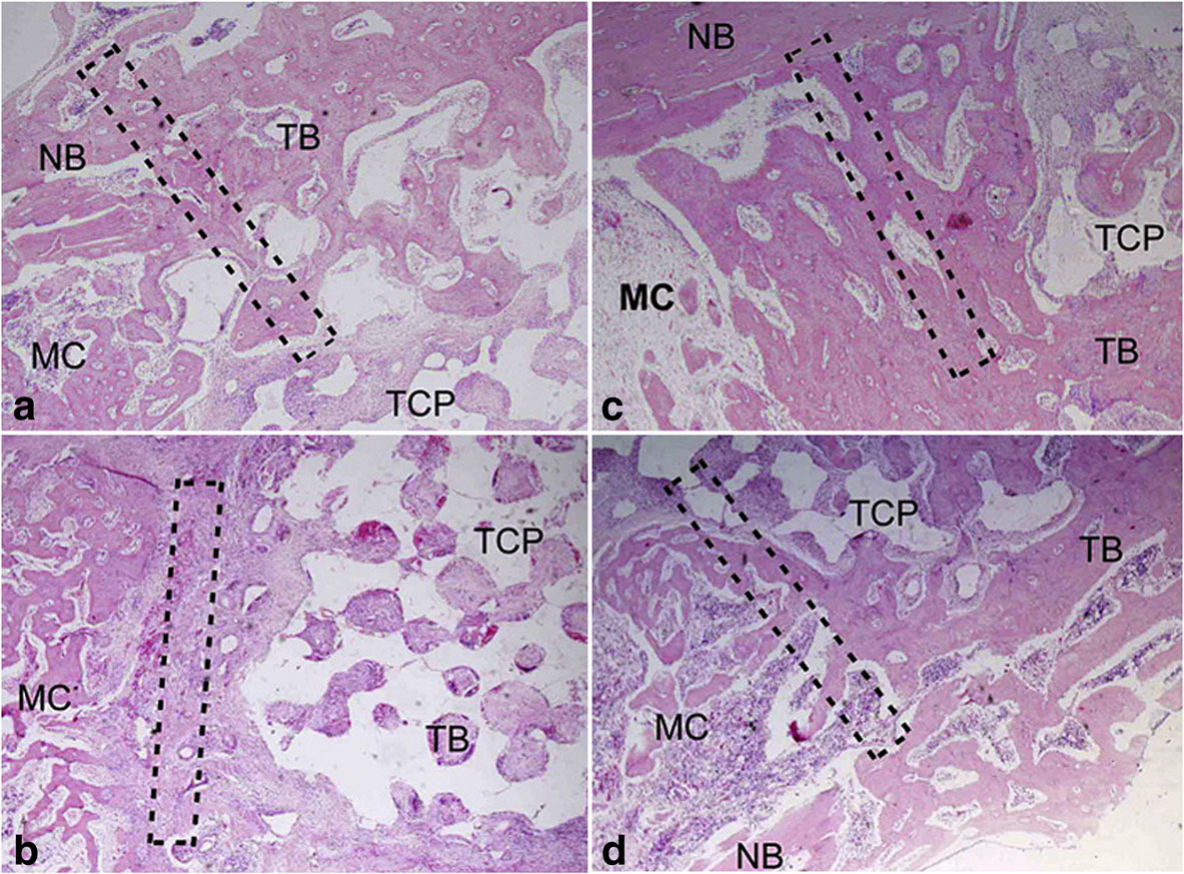
Histological evaluation of interfacial area between the implant and the host bone of PRP group constructs (**a**, **c**) and non-PRP group constructs (**b**, **d**) at 4 weeks (**a**, **b**) and 8 weeks (**c**, **d**) postoperatively. The newly formed bone grew and merged at interfacial area for both groups with implantation period prolonging. However, the amount and rate of the new bone formation of the non-PRP group was less and slower than that of PRP group (HE staining; × 40. TCP, tricalcium phosphate; TB, tissue-engineered bone; NB, native bone; MC, medullary cavity. The dotted rectangles indicate the interfacial area)

Under a light microscope, the micro pores of the implanted constructs in both groups were filled with loose connective tissue at 4 weeks postoperatively. More newly formed bone tissues were deposited in PRP group. With the implantation prolonged, the new bone formation and the degradation of scaffolds were increasingly obvious. At 8 weeks postoperatively, the PRP group demonstrated more extensive bone formation with the degradation of most of the scaffolds compared with the non-PRP group (Fig. 5). Furthermore, the amount of newly formed bone varied in the different locations of implants. New bone formation was more obvious in the peripheral regions of implants than that in the central regions at any time point. The percentage of new bone tissue in the defect region increased with time in both groups. The extent of new bone formation was significantly more obvious in the PRP group than that in the non-PRP group at each time point (*p* < 0.05, Table 4). For the amount of remaining β-TCP and connective tissue, inverse results were found, also reaching statistical significance (*p* < 0.05, Table 4). At the interfacial area between the implant and the host bone, newly formed bone grew and merged for both groups with prolonged implantation period. Although similar histological changes occurred, the amount and rate of new bone formation of the non-PRP group was less and slower than that of the PRP group (Fig. 6).

**Table 4:** Tissue composition by the new bone, β-TCP, and connective tissue of implants (*n* = 6, mean ± SD)

Group	Time	New bone (%)	β-TCP (%)	Connective tissue (%)
Non-PRP group	4 weeks	16.333 ± 2.16	41 ± 3.578	41.333 ± 3.011
PRP group	4 weeks	29.667 ± 1.366*	36 ± 3.847*	36 ± 4.243*
Non-PRP group	8 weeks	29.333 ± 1.75	36.5 ± 1.871	33.833 ± 2.858
PRP group	8 weeks	39.5 ± 1.871*	29.5 ± 1.049*	29.333 ± 2.582*

*p < 0.05 compared with the non-PRP group

## Discussion

The hypothesis for this study was that the introduction of autologous PRP may improve the osteogenic potential of tissue-engineered bones. In vitro study, MTT assay demonstrated that the MSCs in the presence of autologous PRP exhibited excellent proliferation at each time point. Osteogenic differentiation results showed significantly high levels of synthesis of ALP and OC by the MSCs in combination with autologous PRP after 7 and 14 days of culture. No OC was detected for both groups at 1 day of cultivation. This is probably because OC is a late marker of the maintenance of the osteoblastic phenotype. With regard to the bone-forming capacity in vivo, radiographic and histological observation showed that the PRP group constructs accelerated bone healing in rabbit segmental defects of radial diaphyses over the 8-week period, compared with the non-PRP group constructs. The radiographic observation showed that the PRP group produced significantly higher score than the Non-PRP group at each time point. For histological evaluation, significantly higher volume of regenerated bone was found in the PRP group when compared with the non-PRP group at each time point. Given these results, it is clear that the autologous PRP had a stimulative effect on in vitro proliferation and osteogenic differentiation, and in vivo bone formation of MSCs in our experimental setting.

PRP develops a fibrin gel network containing a myriad of growth factors after activation, such as platelet-derived growth factor (PDGF) and transforming growth factor (TGF). All these factors have a stimulating effect on bone defect healing via chemotactic and mitogenic effects on preosteoblastic and osteoblastic cells [7, 8, 24–26]. Thus, the possibility of delivering these matrix elements and growth factors within a bone defect is behind the theory of the use of PRP in bone reconstruction therapy. Since the local application of PRP was proposed by Marx et al. [27] to enhance and accelerate the maturity of corticocancellous grafts to repair continuity defects of the maxillofacial region in 1998, PRP has been widely used in preclinical and clinical applications for regeneration of the bone. However, its use has never produced consistent and reliable results in terms of bone regeneration. The inconsistency of results found in various studies may result from differences in platelet concentration, biology among species, bone defect models and bone grafting materials combined with PRP. It should be noticed that these studies should not be directly compared because the study designs were very different.

Platelet concentration of prepared PRP is one of the key factors that may help in understanding such controversial results about the effectiveness of PRP. Platelet count in PRP may vary according to the preparation technique, ranging from two to several folds above the physiological levels. It has been stated by Weibrich et al. [28] that advantageous biological effects seem to occur when PRP with a platelet concentration of approximately 1,000,000/μl is used. At lower concentrations, the effect is suboptimal, while higher concentrations might have a paradoxically inhibitory effect. It also has been suggested that PRP should achieve a two- to sixfold increase in platelet concentration over baseline [7, 8]. In the current study, PRP platelet count were 1056 ± 106 × 10^3^ platelets/μl, a measured increase of 525 ± 36% from whole blood. Correspondingly, PRP produced 2.64-fold increase in PDGF-AB concentration and 3.54-fold increase in TGF-β1. This may represent an explanation for the positive results of the present study.

With regard to grafting materials combined with PRP, several studies have evaluated the effect of PRP in combination with β-TCP materials. The study from Li H et al. [29] reported that the addition of PRP to β-TCP failed to improve bone healing in an anterior spinal fusion. The reason for the failure of PRP might be the absence of precursor cells. The study by Kasten P et al. [30] found that MSC/TCP failed to profit from the frozen PRP for ectopic bone formation. One possible explanation is that that frozen PRP has weaker osteogenic properties than fresh PRP. Nowadays, there is no data that proves that frozen PRP is equally effective as fresh PRP regarding osteogenesis. The beneficial effect of autologous PRP combined with β-TCP was reported by Long Bi et al. [31], who used a goat tibial cavity defect model.

In the present study, a segmental bone defect model in a rabbit radius was adapted for low regenerative potential and similarity to the clinical situation, which is of great interest for an orthopedic surgeon. Management of large segmental bone defects, particularly in diaphyseal bones, remains a considerable challenge for orthopedic surgeons. In recent years, tissue engineering has provided a promising method for repairing such bone defects [32]. However, the clinical application of these advances in the field of tissue engineering is still limited. Increasing research has found that the key factor contributing to poor repair with a tissue-engineered bone is poor vascularization [33, 34]. Several strategies for enhancing vascularization are currently under investigation. These include modification of the scaffold design, delivery of angiogenic factors, and prevascularization procedure [35]. Vascular endothelial growth factor (VEGF) as angiogenic factors can enhance regional vascularization by altering the responsiveness of local endothelia cells to angiogenic stimuli [36, 37]. Furthermore, VEGF was demonstrated by Street et al. [38] to stimulate bone repair by promoting angiogenesis and bone turnover. When PRP is combined with TCP scaffolds, the platelet can release the VEGF. So the fabricated constructs possess angiogenic factors, which may improve the bone regeneration by increasing vascular growth. This may be one of the reasons for more effective repair of segmental bone defect models. The limitation of the current experimental design is that the blood vessel formation of tissue-engineered bone constructs was not investigated. This should be addressed in future studies.

At present, it is recognized that growth factors regulate fracture healing and physiological remodeling by inducing chemotaxis, differentiation, proliferation, and synthetic activity of bone cells. However, the combinations, concentrations, and application time points of various growth factors in reparative processes were poorly understood. Our findings support the use of autologous PRP as an easy and physiological way of application for growth factors in natural composition to improve bone regeneration. A combination of MSCs with PRP on biomechanically stable β-TCP scaffolds fulfills the requirements of ideal bone graft substitutes: progenitor cells for osteogenesis, scaffold material for osteoconduction, and growth factors for osteoinduction. All these may explain the success of fabricated tissue-engineered bone observed in our experimental setting. Furthermore, the advantages of autologous PRP also include its safety and its availability in an easy-to-develop manner.

## Conclusions

Our study findings support the osteogenic capacity of autologous PRP. The results indicate that the use of autologous PRP is a simple and effective way to provide osteoinduction and improve bone regeneration for tissue-engineered bone reconstruction.

## Abbreviations

ALP: Alkaline phosphatase
MSC: Marrow mesenchymal stem cell
OC: Osteocalcin
PDGF: Platelet-derived growth factor
PRP: Platelet-rich plasma
TGF: Transforming growth factor
β-TCP: Beta-tricalcium phosphate

## References

[1] Bauer TW, Muschler GF (2000). Bone graft materials. An overview of the basic science. Clin Orthop Relat Res.

[2] Schroeder JE, Mosheiff R (2011). Tissue engineering approaches for bone repair: concepts and evidence. Injury.

[3] Drosse I, Volkmer E, Capanna R, De Biase P, Mutschler W, Schieker M (2008). Tissue engineering for bone defect healing: an update on a multi-component approach. Injury.

[4] Pountos I, Giannoudis PV (2005). Biology of mesenchymal stem cells. Injury.

[5] Fujibayashi S, Shikata J, Tanaka C, Matsushita M, Nakamura T (2001). Lumbar posterolateral fusion with biphasic calcium phosphate ceramic. J Spinal Disord.

[6] Kasten P, Beyen I, Niemeyer P, Luginbühl R, Bohner M, Richter W (2008). Porosity and pore size of beta-tricalcium phosphate scaffold can influence protein production and osteogenic differentiation of human mesenchymal stem cells: an in vitro and in vivo study. Acta Biomater.

[7] Marx RE (2004). Platelet-rich plasma: evidence to support its use. J Oral Maxillofac Surg.

[8] Intini G (2009). The use of platelet-rich plasma in bone reconstruction therapy. Biomaterials.

[9] Kitoh H, Kitakoji T, Tsuchiya H, Katoh M, Ishiguro N (2007). Transplantation of culture expanded bone marrow cells and platelet rich plasma in distraction osteogenesis of the long bones. Bone.

[10] Simon Z, Friedlich J (2006). The use of autogenous bone grafting with platelet-rich plasma for alveolar ridge reconstruction: a clinical report. J Calif Dent Assoc.

[11] Aghaloo TL, Moy PK, Freymiller EG (2002). Investigation of platelet-rich plasma in rabbit cranial defects: a pilot study. J Oral Maxillofac Surg.

[12] Froum SJ, Wallace SS, Tarnow DP, Cho SC (2002). Effect of platelet-rich plasma on bone growth and osseointegration in human maxillary sinus grafts: three bilateral case reports. Int J Periodontics Restorative Dent.

[13] Choi BH, Im CJ, Huh JY, Suh JJ, Lee SH (2004). Effect of platelet-rich plasma on bone regeneration in autogenous bone graft. Int J Oral Maxillofac Surg.

[14] Gerard D, Carlson ER, Gotcher JE, Jacobs M (2006). Effects of platelet-rich plasma on the healing of autologous bone grafted mandibular defects in dogs. J Oral Maxillofac Surg.

[15] Karageorgiou V, Kaplan D (2005). Porosity of 3D biomaterial scaffolds and osteogenesis. Biomaterials.

[16] Kamitakahara M, Ohtsuki C, Miyazaki T (2008). Behavior of ceramic biomaterials derived from tricalcium phosphate in physiological condition. J Biomater Appl.

[17] Liu H, Li H, Cheng W, Yang Y, Zhu M, Zhou C (2006). Novel injectable calcium phosphate/chitosan composites for bone substitute materials. Acta Biomater.

[18] Ishida K, Kuroda R, Miwa M, Tabata Y, Hokugo A, Kawamoto T (2007). The regenerative effects of platelet-rich plasma on meniscal cells in vitro and its in vivo application with biodegradable gelatin hydrogel. Tissue Eng.

[19] Tajima N, Sotome S, Marukawa E, Omura K, Shinomiya K (2007). A three-dimensional cell-loading system using autologous plasma loaded into a porous β-tricalcium-phosphate block promotes bone formation at extraskeletal sites in rats. Mater Sci Eng C.

[20] Wang H, Li Y, Zuo Y, Li J, Ma S, Cheng L (2007). Biocompatibility and osteogenesis of biomimetic nano-hydroxyapatite/polyamide composite scaffolds for bone tissue engineering. Biomaterials.

[21] Jiang T, Abdel-Fattah WI, Laurencin CT (2006). In vitro evaluation of chitosan/poly(lactic acid-glycolic acid) sintered microsphere scaffolds for bone tissue engineering. Biomaterials.

[22] Niemeyer P, Szalay K, Luginbühl R, Südkamp NP, Kasten P (2010). Transplantation of human mesenchymal stem cells in a non-autogenous setting for bone regeneration in a rabbit critical-size defect model. Acta Biomater.

[23] Yang CY, Simmons DJ, Lozano R (1994). The healing of grafts combining freeze-dried and demineralized allogeneic bone in rabbits. Clin Orthop Relat Res.

[24] Fiedler J, Roderer G, Gunther KP, Brenner RE (2002). BMP-2, BMP-4, and PDGF-bb stimulate chemotactic migration of primary human mesenchymal progenitor cells. J Cell Biochem.

[25] Baylink DJ, Finkelman RD, Mohan S (1993). Growth factors to stimulate bone formation. J Bone Miner Res.

[26] Bostrom MP, Saleh KJ, Einhorn TA (1999). Osteoinductive growth factors in preclinical fracture and long bone defects models. Orthop Clin North Am.

[27] Marx RE, Carlson ER, Eichstaedt RM, Schimmele SR, Strauss JE, Georgeff KR (1998). Platelet-rich plasma: growth factor enhancement for bone grafts. Oral Surg Oral Med Oral Pathol Oral Radiol Endod.

[28] Weibrich G, Hansen T, Kleis W, Buch R, Hitzler WE (2004). Effect of platelet concentration in platelet-rich plasma on peri-implant bone regeneration. Bone.

[29] Li H, Zou X, Xue Q, Egund N, Lind M, Bünger C (2004). Anterior lumbar interbody fusion with carbon fiber cage loaded with bioceramics and platelet-rich plasma. An experimental study on pigs. Eur Spine J.

[30] Kasten P, Vogel J, Luginbühl R, Niemeyer P, Weiss S, Schneider S (2006). Influence of platelet-rich plasma on osteogenic differentiation of mesenchymal stem cells and ectopic bone formation in calcium phosphate ceramics. Cells Tissues Organs.

[31] Bi L, Cheng W, Fan H, Pei G (2010). Reconstruction of goat tibial defects using an injectable tricalcium phosphate/chitosan in combination with autologous platelet-rich plasma. Biomaterials.

[32] Dumic-Cule I, Pecina M, Jelic M, Jankolija M, Popek I, Grgurevic L (2015). Biological aspects of segmental bone defects management. Int Orthop.

[33] Nakasa T, Ishida O, Sunagawa T, Nakamae A, Yasunaga Y, Agung M (2005). Prefabrication of vascularized bone graft using a combination of fibroblast growth factor-2 and vascular bundle implantation into a novel interconnected porous calcium hydroxyapatite ceramic. J Biomed Mater Res A.

[34] Kawamura K, Yajima H, Ohgushi H, Tomita Y, Kobata Y, Shigematsu K (2006). Experimental study of vascularized tissue-engineered bone grafts. Plast Reconstr Surg.

[35] Rouwkema J, Rivron NC, van Blitterswijk CA (2008). Vascularization in tissue engineering. Trends Biotechnol.

[36] Cao L, Arany PR, Wang YS, Mooney DJ (2009). Promoting angiogenesis via manipulation of VEGF responsiveness with notch signaling. Biomaterials.

[37] Huang YC, Kaigler D, Rice KG, Krebsbach PH, Mooney DJ (2005). Combined angiogenic and osteogenic factor delivery enhances bone marrow stromal cell-driven bone regeneration. J Bone Miner Res.

[38] Street J, Bao M, de Guzman L, Bunting S, Peale FV, Ferrara N (2002). Vascular endothelial growth factor stimulates bone repair by promoting angiogenesis and bone turnover. Proc Natl Acad Sci U S A.

